# A molecular biomarker for prediction of clinical outcome in children with ASD, constipation, and intestinal inflammation

**DOI:** 10.1038/s41598-019-42568-1

**Published:** 2019-04-12

**Authors:** Stephen J. Walker, Carl D. Langefeld, Kip Zimmerman, Marshall Z. Schwartz, Arthur Krigsman

**Affiliations:** 1 Wake Forest University Health Sciences, Winston-Salem, North Carolina USA; 2 Wake Forest Institute for Regenerative Medicine, Winston-Salem, North Carolina USA; 3 Department of Biostatistical Sciences, Division of Public Health Sciences, Wake Forest School of Medicine, Winston-Salem, North Carolina USA; 4 Pediatric Gastroenterology Resources of New York and Texas, Austin, Texas USA

## Abstract

In children with autism spectrum disorder (ASD) who present to the gastroenterologist with chronic constipation on a background of colonic inflammation, we have identified two distinct clinical subtypes: (1) patients who experience a sustained state of GI symptomatic remission while on maintenance anti-inflammatory therapy (*fast responders*) and, (2) those with recurrent right-sided fecal loading requiring regular colon cleanouts during treatment for enterocolitis (*slow responders*). We hypothesized that a detailed molecular analysis of tissue from the affected region of the colon would provide mechanistic insights regarding the fast versus slow response to anti-inflammatory therapy. To test this, ascending colon biopsy tissues from 35 children with ASD (20 *slow responders* and 15 *fast responders*) were analyzed by RNAseq. Hierarchical cluster analysis was performed to assign samples to clusters and gene expression analysis was performed to identify differentially expressed transcripts (DETs) between samples within the clusters. Significant differences were found between the two clusters with *fast responder*-predominant cluster showing an upregulation of transcripts involved in the activation of immune and inflammatory response and the *slow responder*-predominant cluster showing significant over-representation of pathways impacting colonic motility (e.g. genes involved in tryptophan and serotonin degradation and mitochondrial dysfunction). Regression analysis identified a single long non-coding RNA that could predict cluster assignment with a high specificity (0.88), sensitivity (0.89) and accuracy (0.89). Comparison of gene expression profiles in the ascending colon from a subset of patients with ASD, chronic right-sided fecal loading constipation and a slow versus fast response to therapy has identified molecular mechanisms that likely contribute to this differential response following the primary therapeutic intervention (i.e. treatment for colonic inflammation with brief induction immunosuppression followed by maintenance non-steroidal anti-inflammatory therapy). Importantly, we have identified a transcript that, if validated, may provide a biomarker that can predict from the outset which patients will be slow responders who would benefit from an alternate therapeutic strategy in treating their constipation.

## Introduction

Gastrointestinal (GI) symptoms are common in children with autism spectrum disorder (ASD)^1^ and have been shown to be associated with more severe deficits in ASD core domains related to cognition and behavior^2–6^. Of the GI symptoms identified most commonly in children with ASD (abdominal pain, diarrhea and constipation), chronic constipation is the symptom most frequently reported by parents as being especially problematic^7^.

Although prevalence estimates vary, it has been reported that approximately 1 in 5 children had constipation at the time of their initial diagnostic evaluation for ASD^8^ and that by 20 years of age, 1 in 3 children with ASD had sought medical assistance for constipation^9^. Constipation in children with ASD has also been found to be associated with increased emergency department (ED) visits and inpatient admissions^10^. Importantly, constipation in younger children (age 5–12 years) was far more likely to be the presenting symptom of the ED visit in children with ASD compared to typically developing (TD) children.

In our experience treating more than 1500 GI-symptomatic children with ASD, the predominant pattern of ASD-associated constipation is characterized by infrequent stools (two or less per week) that are semi-formed or formed in texture (Bristol Type III-IV), accompanied by fecal incontinence, anal leakage, and stool retention (evidenced in abdominal radiographs). In contrast to pediatric functional constipation seen in TD children where the child is voluntarily attempting to withhold stool, parents of children with ASD often describe their child as making clear efforts to pass stool, but without success. In children with ASD, who are often poorly or non-verbally communicative, these efforts are apparent through observation of the child who is displaying a variety of Valsalva-type maneuvers.

Diarrhea in children with ASD is similar to diarrhea in TD children when defined in terms of texture (Bristol Stool Type V-VII) and frequency (three or more times per day)^7^. Diarrhea in ASD is characteristically extremely malodorous, light colored (beige or yellow), and typically contains significant amounts of undigested food^11^. The authors’ unpublished experience is that diarrheal stools are often passed with excessive effort and straining; parents and caretakers often express surprise at the degree of effort needed to pass such loose stools.

Finally, a pattern of alternating diarrhea and constipation may also occur wherein the child experiences days or weeks of predominantly diarrhea alternating with days or weeks of predominantly constipation^7^. A review of 143 patients with ASD and chronic GI symptoms found similar frequencies in underlying intestinal inflammation in all three of these groups (i.e. constipation, diarrhea, and alternating constipation and diarrhea)^11^.

One of the more unusual patterns of constipation we commonly encounter in children with ASD and associated ASD enterocolitis is characterized by fecal loading (i.e. “soft stool constipation”) and is apparent in abdominal radiographs. The fecal loading at presentation, while typically pancolonic, often demonstrates right-sided predominance in the cecum and ascending colon, with less loading distally. The unusual right-sided nature of this presentation and its predominance in the setting of ASD and intestinal inflammation distinguishes it from classic slow transit constipation. This distinction is best appreciated in the context of the variable response to the standard therapeutic approach of laxative administration. In many patients, post-cleanout abdominal radiographs following therapeutic bowel cleansing with osmotic and irritant oral laxatives and enemas/suppositories demonstrate successful emptying of the transverse and descending colon and rectum but with a persistence of fecal loading in the cecum and ascending colon. In these patients (i.e. *slow responders; ~*30% of those exhibiting chronic constipation), regularly scheduled bowel cleanouts are often needed, in conjunction with ongoing oral anti-inflammatory agents, to prevent persistent symptomatic constipation and the myriad behavioral issues associated with chronic gastrointestinal symptoms in this population^12,13^. The prevalence of this *slow responder* phenotype appears to increase with age.

These observations suggest a localized dysmotility of the cecum and ascending (right) colon in these patients. Importantly, this pattern of hypomotility presents on a background of enterocolitis. Based on our clinical observations, we hypothesized that GI-symptomatic children with ASD and fecal loading may harbor evidence of agents (e.g. chemical, infectious, or inflammatory) underlying the right-sided colonic dysmotility. Furthermore, clues to the etiology of this phenomenon may be revealed via molecular analysis of mucosal and submucosal colonic biopsy tissue from these patients.

To test this hypothesis, we performed molecular analysis of colonic tissue from two groups of GI-symptomatic children with ASD and chronic constipation on a background of enterocolitis. The presenting chronic refractory constipation in all patients, prior to visiting the gastroenterologist, had not responded to laxative therapy alone. In the first group, *fast responding chronic constipation*, patients experienced resolution of their presenting symptoms including constipation and, with ongoing anti-inflammatory (maintenance) therapy for underlying intestinal inflammation, no longer had a clinical course indicative of chronic constipation. Patients in the second group, *slow responding chronic constipation (with fecal loading*), had radiographic evidence of persistent localized fecal loading in the cecum and ascending colon and a clinical course marked by recurrent symptomatic fecal loading requiring regular colon cleanouts despite ongoing anti-inflammatory therapy for intestinal inflammation.

The latter “slow responder” group is noteworthy in that although their other presenting GI symptoms (e.g. diarrhea, abdominal pain, abdominal distention, abnormal stool color and odor, etc.) did respond to anti-inflammatory therapy targeting their mucosal inflammation, one symptom - recurring right sided hypomotility with localized right sided fecal loading - persisted.

The goal of this study was to provide mechanistic context for this divergence in clinical course by comparing the gene expression profiles within the right colonic mucosal and submucosal tissues from these two groups of otherwise similar patients. To our knowledge, this is the first report investigating the molecular basis of right-sided colonic dysmotility (i.e. fecal loading) in GI-symptomatic children with ASD.

## Results

**Figure 1:**
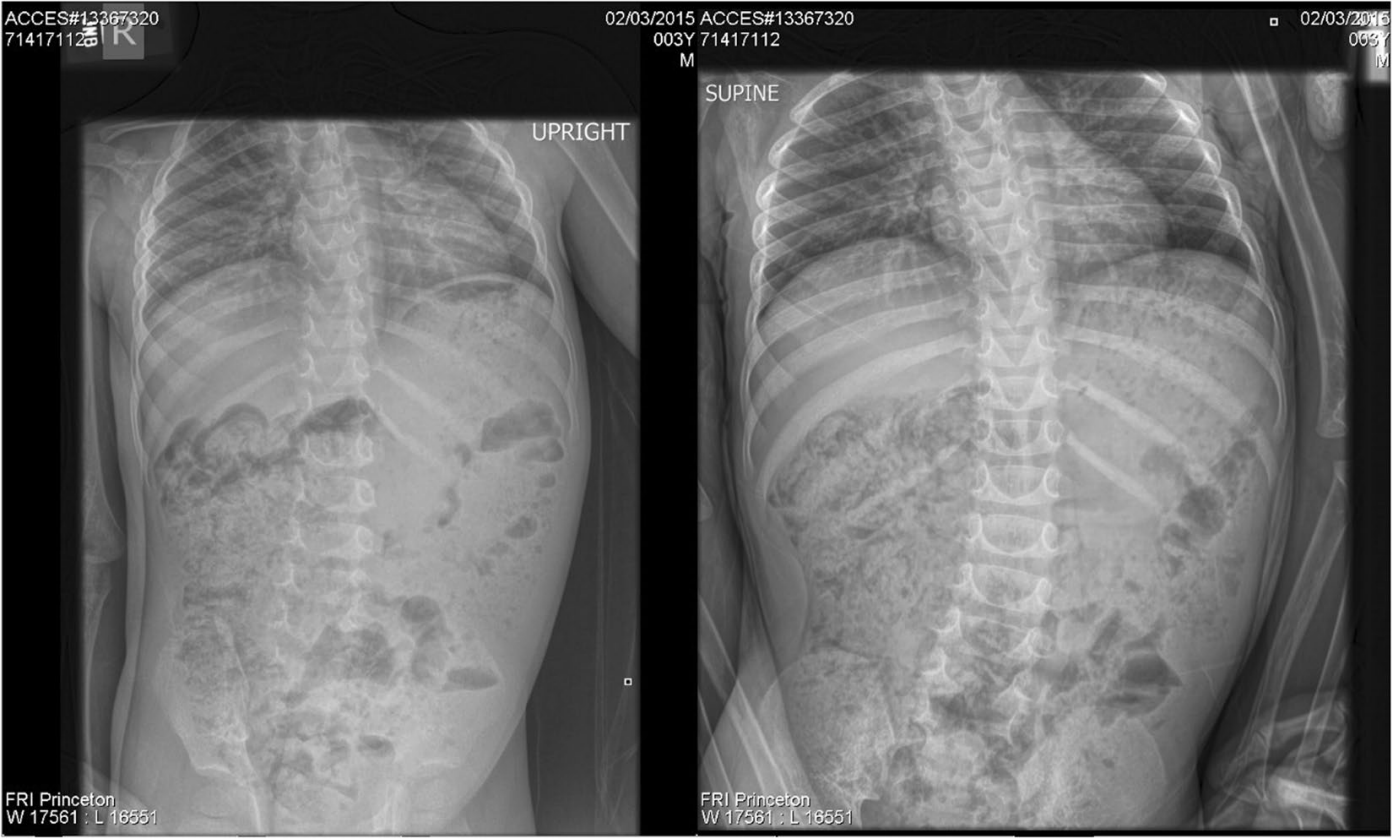
Abdominal radiograph of 3 year old boy with autism and right-sided colonic fecal loading with colonic distention.

The first group (i.e. *slow* responders) was comprised of samples from 20 GI-symptomatic children with ASD (16M:4F; 3.6–15.2 years old) that displayed persistent right-sided fecal loading (Fig. 1) while the second group (*fast* responder) consisted of samples from an additional 15 GI-symptomatic children with ASD (13M:2F; 2.1–17.9 years old) (Table 1). Although both groups contained a comparable number of males and females (p = 0.6804), the slow responders tended to be older (p = 0.0217).

**Table 1:** Study participant demographics.

	Slow Response	Fast Response
**Number**	20	15
**Age (years)**		
Mean (SD)	9.0 (3.81)	5.8 (3.61)
Range	3.6–15.2	2.1–17.9
**Sex**		
Male (%)	16 (80)	13 (87)
Female (%)	4 (20)	2 (13)

### All samples

**Figure 2:**
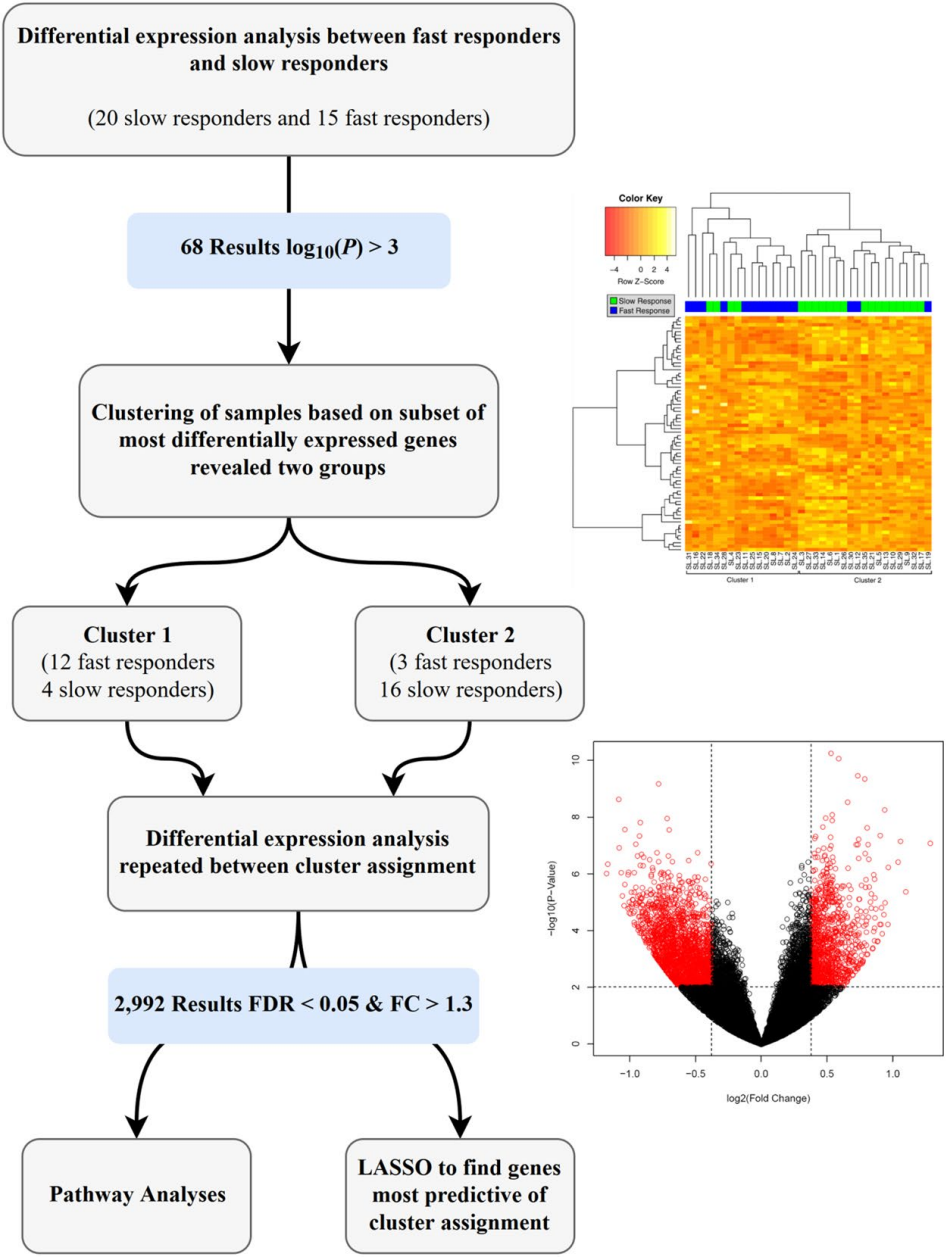
Flow diagram depicting data analysis scheme. Top Right: Heatmap representing all study samples. Hierarchical cluster analysis was computed using an inter-individual similarity matrix of Euclidean distances between the normalized expression values for each of the samples. This analysis resulted in two distinct clusters (as indicated). Bottom Right: Volcano plot representing the distribution of the 2992 differentially expressed genes between *cluster 1* and *cluster 2*.

Figure 2 provides an outline of the data analysis scheme. The initial differential expression analysis yielded 68 DETs (p < 0.001) between slow and fast response groups. The DET list featured 29 up-regulated and 39 down-regulated transcripts (Additional File 1). IPA analysis of these differentially expressed transcripts revealed that one of the top physiological system development and function categories most over-represented in this set of genes was *digestive system development* (p = 4.13 × 10^−02^–1.91 × 10^−03^) and the top disease and bio functions categories were *metabolic* disease (p = 4.86 × 10^−02^–3.34 × 10^−05^), *developmental disorder* (p = 3.76 × 10^−02^–3.56 × 10^−05^), *neurological disease* (p = 4.84 × 10^−02^–3.56 × 10^−05^), and *organismal injury and abnormalities* (p = 5.00 × 10^−02^–3.56 × 10^−05^).

Next, unsupervised hierarchical cluster analysis of these 68 DETs and the final dendogram, overlaid with a heatmap of the normalized expression values for each gene, revealed two clusters of patients (Fig. 2 – heatmap). The first cluster contained mostly fast responders (12 fast, 4 slow) while the second cluster contained mostly slow responders (3 fast, 16 slow; P = 0.0004). This partitioning is important as the hierarchical cluster analysis and related dendogram were computed agnostic to slow/fast response.

### Cluster 1 versus Cluster 2

Since cluster 1 and cluster 2 (Fig. 2 – heatmap) represent significant subgroups and contain the majority of fast responders (*cluster 1*; fast response chronic constipation) and slow responders (*cluster 2*; slow response chronic constipation), differential gene expression analysis was performed contrasting these two clusters and identified 2,992 total DETs (Fig. 2 – volcano plot; i.e., 1960 up-regulated in *cluster 1* compared to *cluster 2*; 1031 up-regulated in *cluster 2* compared to *cluster 1*) that met an average read count threshold >5, FDR adjusted p-value < 0.05 and a fold change of 1.3 (or its inverse). The top five *canonical pathways*, *physiologic functions*, and *diseases and biological functions* significantly over-represented by this gene set, as identified by the Ingenuity Pathway Analysis (IPA) software, are reported in Table 2.

**Table 2:** Ingenuity Pathway Analysis results.

Pathways	p-value – range
Th1 and Th2 Activation Pathway	1.65E-25
Th2 Pathway	1.10E-20
Th1 Pathway	1.11E-20
iCOS-iCOSL Signaling in T Helper Cells	5.45E-117
Altered T Cell and B Cell Signaling	608E-16
**Physiologic Function**	
Hematological System Development and Function	6.19E-14–1.80E-73
Tissue Morphology	6.04E-14–1.80E-73
Lymphoid Tissue Structure and Development	6.08E-14–1.85E-72
Immune Cell Trafficking	6.19E-14–9.05E-67
Hematopoiesis	6.08E-14–7.80E-63
**Top Diseases and Biological Functions**	
Cancer	5.72E-14–6.44E-71
Organismal Injury and Abnormalities	6.04E-14–6.44E-71
Inflammatory Response	2.58E-14–9.05E-67
Dermatological Diseases and Conditions	2.46E-14–1.75E-65
Gastrointestinal Disease	5.38E-15–2.27E-49

Top five over-represented biological pathways and functions from a comparison of differential gene expression (N = 2992 DETs) between *cluster 1* (mostly fast responders) and *cluster 2* (mostly slow responders) in right colonic tissue from children with ASD and *slow response* versus *fast response* constipation.

### Cluster 1

**Figure 3:**
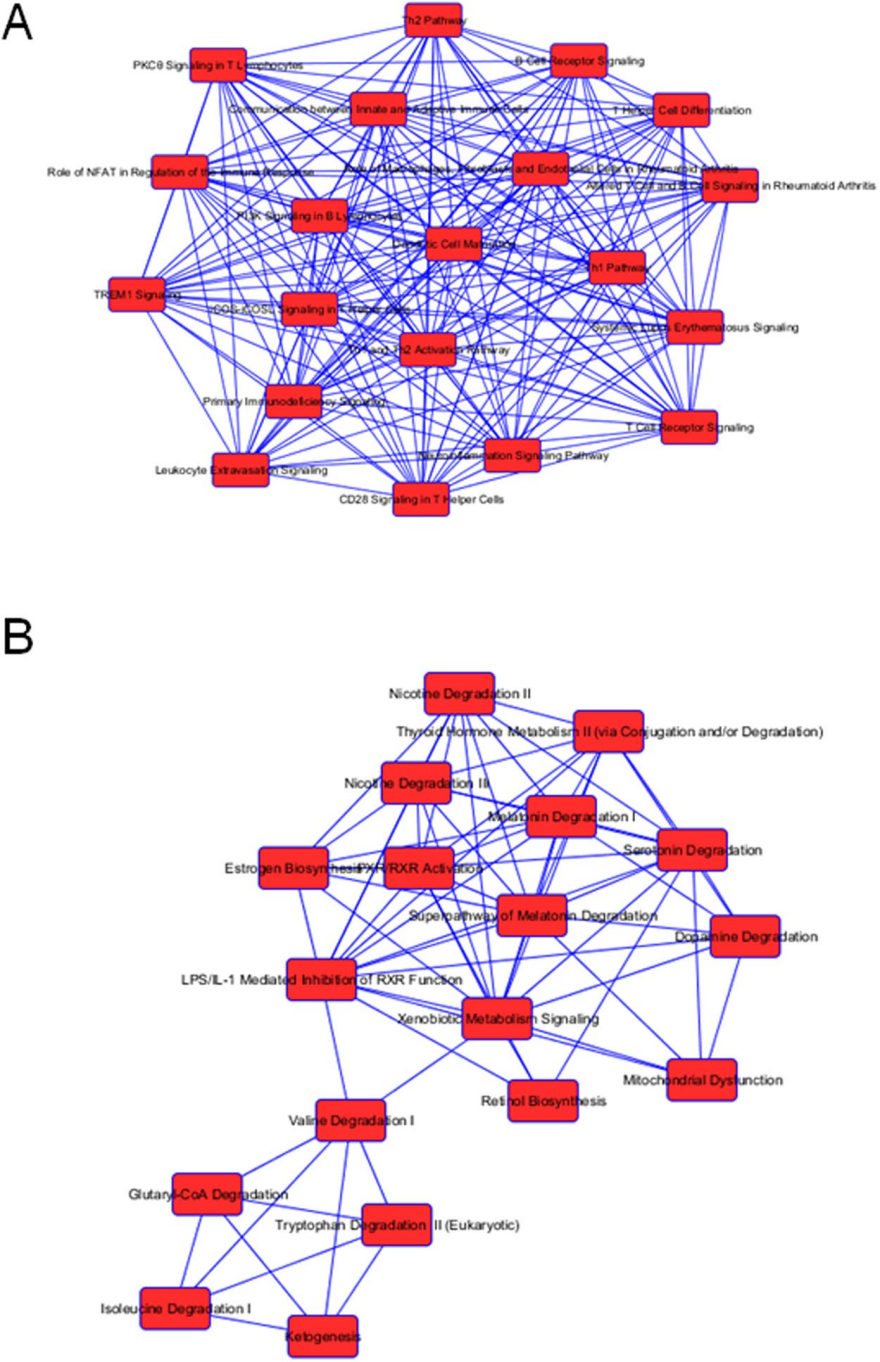
Canonical pathway network analysis. (**A**) Top pathways that contain a majority of upregulated genes in the fast responder group. (**B**) Top pathways that contain a majority of upregulated genes in the slow responder group. Blue lines indicate overlapping (shared) genes in one or more pathways. Numbers = p-value for gene representation in a given pathway from this dataset.

The top five canonical pathways listed in Table 2 involve the cellular and humoral immune responses, cytokine signaling, pathogen-influenced signaling and disease-specific pathways. The vast majority of DETs populating each of these pathways are significantly up-regulated in *cluster 1* (i.e. the fast responder samples). Moreover, dozens of additional biological pathways involved in immune and inflammatory responses and signaling pathways that were revealed in the IPA analysis are primarily populated with transcripts that are expressed at significantly higher levels in patient samples from the fast responder group. The top pathways (i.e. those with the most significant p value) for each of the two clusters, wherein the *majority* of DETS are expressed at a higher level in either one cluster or the other, are listed in Table 3 and illustrated as networks in Fig. 3A. The *top diseases and biological functions* significantly over-represented by this gene set are *inflammatory response* (1.19E-18–4.73E-92) and *immunological disease* (3.08E-18–5.22E-69).

**Table 3:** Top twenty canonical pathways generated in IPA wherein the majority of differentially-expressed transcripts are up-regulated in one of the two clusters.

Up-Regulated in Chronic (Fast Response) Constipation	p-value
Th1 and Th2 Activation Pathway	8.85E-35
Th1 Pathway	2.86E-28
Th2 Pathway	9.79E-28
iCOS-iCOSL Signaling in T Helper Cells	2.32E-22
Altered T Cell and B Cell Signaling in Rheumatoid Arthritis	5.22E-21
Communication between Innate and Adaptive Immune Cells	4.22E-20
T Helper Cell Differentiation	5.13E-19
Primary Immunodeficiency Signaling	6.65E-19
CD28 Signaling in T Helper Cells	1.75E-17
Dendritic Cell Maturation	3.31E-17
T Cell Receptor Signaling	3.27E-15
Systemic Lupus Erythematosus Signaling	1.88E-14
Leukocyte Extravasation Signaling	1.09E-13
Role of Macrophages, Fibroblasts and Endothelial Cells in Rheumatoid Arthritis	1.17E-13
Role of NFAT in Regulation of the Immune Response	1.24E-13
TREM1 Signaling	1.36E-13
B Cell Receptor Signaling	3.71E-13
PKCθ Signaling in T Lymphocytes	4.06E-13
Neuroinflammation Signaling Pathway	1.42E-11
PI3K Signaling in B Lymphocytes	2.18E-11
**Up-regulated in Chronic (Slow Response) Constipation**	
Superpathway of Melatonin Degradation	1.72E-13
Melatonin Degradation I	6.32E-12
Serotonin Degradation	1.20E-11
Nicotine Degradation II	7.20E-11
Nicotine Degradation III	8.95E-10
LPS/IL-1-Mediated Inhibition of RXR Function	9.77E-10
Thyroid Hormone Metabolism II	3.64E-09
Xenobiotic Metabolism Signaling	2.17E-08
PXR/RXR Activation	3.79E-06
Glutaryl-CoA Degradation	8.44E-05
α-tocopherol Degradation	1.09E-04
Ketogenesis	1.53E-04
Valine Degradation I	1.57E-0.4
Estrogen Biosynthesis	2.12E-04
Retinol Biosynthesis	2.48E-04
Urea Cycle	5.22E-04
Isoleucine Degradation I	6.64E-04
Mitochondrial Dysfunction	7.59E-04
Dopamine Degradation	8.02E-04
Bupropion Degradation	8.19E-04

### Cluster 2

There were a large number of biological pathways wherein the vast majority of the DETS were expressed at a significantly higher level in the slow response samples compared to the fast responders. The top pathways containing a majority of over-expressed transcripts in the slow response group (*cluster 2*) reflected a very different set of biological themes than those in *cluster 1* (Table 3; Fig. 3B). The pathways are involved in degradation (serotonin, melatonin, nicotine, glutaryl-CoA, alpha-tocopherol, carboxylates, valine and isoleucine), pathogen influenced signaling, metabolism (thyroid hormone and xenobiotics), biosynthesis (estrogen and retinol) and mitochondrial dysfunction, among others (Table 3). Of particular interest, the mitochondrial dysfunction pathway contains 16 DETs from this comparison, 14 of which are upregulated in the slow response constipation samples. In contrast to *cluster 1*, two of the *top diseases and biological functions* significantly over-represented by this *cluster 2* gene set are *gastrointestinal disease* (8.36E-03–1.15E-20) and *metabolic disease* (7.72E-03–5.71E-11).

### Predicting Cluster Assignment

**Figure 4:**
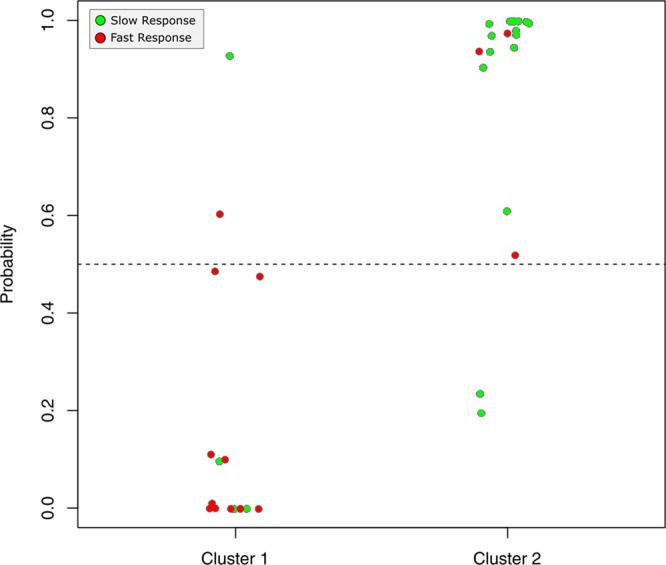
Predictive ability of XXbac.B476C20.9. Using a leave-one-out approach, a single long noncoding RNA Xxbac.B476C20.9 was used to predict where samples would cluster.

The DETs were able to predict cluster assignment well. Specifically, the misclassification error rate of *clusters 1 and 2* from the LASSO regression model was minimized at λ = 0.235 and identified one gene (Xxbac.B476C20.9) whose gene expression profile best predicted sample assignment to *clusters 1 and 2* (Fig. 4). The long noncoding RNA Xxbac.B476C20.9 is known to be highly expressed almost exclusively in the human colon and ileum, compared to all other tissues (Additional File 2). The predicted cluster assignments using the leave-one-out strategy (see Methods) performed quite well when applied to all 35 individuals. The sensitivity (sensitivity = 0.88), specificity (specificity = 0.89), and kappa (kappa = 0.77) statistics all reflect a good strength of agreement between prediction and actual assignments. The area under the receiver operator characteristic curve (AUC) was 0.9605.

## Discussion

All of the patients in this study had a similar clinical presentation of chronic constipation (most often in association with additional gastrointestinal symptoms), and ultimately a diagnosis of intestinal inflammation. In addition, patients segregated into two distinct groups based upon their dissimilar response to an identical therapeutic regimen. Patients in one group experienced rapid resolution of their symptoms of chronic constipation and other presenting GI symptoms following anti-inflammatory therapy and minimal, if any, use of an occasional laxative medications. In contrast, patients in the second group experienced ongoing right-sided fecal loading requiring regularly scheduled bowel clean-outs for an extended period of time (typically 12–36 months), despite complete resolution of other inflammation-associated presenting gastrointestinal symptoms. To date, there is no definitive physiologic explanation for this unusual presentation of persistent right-sided fecal loading in children with ASD, nor any generally accepted therapeutic regimen to manage the patients exhibiting the *slow responder* phenotype. These observations, in a large subset of children with ASD, chronic constipation, and associated GI symptoms, are most consistent with a localized dysmotility within the cecum and ascending colon, of unknown etiology. We suggest that this apparent dysmotility contributes to the ongoing GI symptom complex and the associated behavioral and cognitive deficits following resolution of their inflammatory-mediated symptoms.

Results from gene expression profiling of right colonic mucosal biopsy specimens in these two groups of patients with ASD provide further insight into both the underlying pathophysiology as well as the divergence of their clinical responses in terms of their presenting constipation. Compared to slow responders, gene expression patterns in the fast responders were indicative of significant over-expression of large groups of interrelated transcripts. This general over expression involved activation of the T cell receptor mediated immune response (both TH1 and TH2) and an overall inflammatory response, including dozens of related signaling pathways. This includes pathway signaling of iCOS-iCOSL, T cell receptor, leukocyte extravasation, TREM1, NF-kappaB, and Toll-like receptor. In the context of the broader ASD inflammatory bowel disease (IBD)-like syndrome previously described^11,14–20^, this immune signaling and inflammatory mucosal gene expression profile have been reported previously in both adult and pediatric IBD^21–25^, and specifically in GI-symptomatic children with ASD^26–28^.

Importantly, the finding of this GI mucosal pro-inflammatory gene expression profile, prior to treatment, is entirely consistent with the observed clinical resolution of a variety of associated presenting GI symptoms following treatment with corticosteroids and anti-inflammatory pharmaceuticals. As previously noted, the presenting chronic refractory constipation in *all* patients, prior to visiting the gastroenterologist, did not respond to laxative therapy alone. Following the initiation of treatment with anti-inflammatory medication, constipation in the fast responder group, but not the slow responders, resolved in tandem with their other presenting GI symptoms (e.g. diarrhea, distention, and abdominal pain). This suggests that the predominantly chronic constipation in fast responders is not only *related* to the inflammatory status of the right colon but is likely a direct *consequence* of this colonic inflammation.

Although resolution of the associated GI symptoms was achieved with anti-inflammatory pharmaceuticals in the slow responder group, the localized right sided fecal loading persisted, suggesting that a regional overriding pathologic process is occurring in this patient cluster that prevents the normalization of motility following anti-inflammatory therapy. Clues as to the nature of this phenomenon are provided by the predominant gene expression profile of the slow responder cluster. A majority of DETs in pathways impacting colonic motility were *over-expressed* and include: (1) degradation of tryptophan, serotonin and melatonin, (2) activation of pregnane X receptor (PXR), the retinoid X receptor (RXR), and farnesoid X receptor (FXR) receptors, (3) ketogenesis, ketolysis, oxidative phosphorylation, and mitochondrial dysfunction.

A common theme in the pathways shown to have significant upregulation in the slow responder cluster was biomolecule degradation pathways (e.g. amino acids, hormones, and carboxylates). Among the amino acid degradation pathways in this group, tryptophan degradation may be especially significant. The metabolic fate of the essential amino acid tryptophan (TRP) has important implications for gastrointestinal disease. For example, serum tryptophan levels have been found to be significantly lower in patients with IBD than in controls, suggesting a high activity of tryptophan degradation, which was a key finding in the current study, in patients with active IBD^29^. In individuals with ASD, TRP insufficiency has emerged as a possible biomarker for the disorder itself ^30^. Further candidate contributors to ASD are the melatonin and kynurenine metabolic pathways for which TRP is also the precursor^31^. Serotonin (5-hydroxytryptamine; 5-HT), a neurotransmitter synthesized from TRP, is important in the central nervous system (CNS) but also in the enteric nervous system (ENS). In the gastrointestinal tract 5-HT has diverse functions that include regulating both motility and visceral sensitivity^32^. In the slow responder cluster of patients, there was a significant upregulation of transcripts in each of the metabolic *degradation* pathways for tryptophan, serotonin, and melatonin, suggesting that TRP insufficiency (and therefore 5-HT insufficiency) may be an important factor in the sustained hypomotility seen in this patient cohort.

A second theme that has emerged from the molecular data in the slow responder group involves the overexpression of activation pathways for the PXR, RXR, and FXR receptors. The PXR/RXR heterodimer plays a crucial role in xenobiotic and drug metabolism, as well as in bile acid biosynthesis and modification, gluconeogenesis, and lipid metabolism. Moreover, in a study that used microarray and pathway analysis to examine the effect of dietary curcumin and rutin on colonic inflammation, the data suggested that the reduction in colonic inflammation seen in a mouse model of Crohn’s disease may be attributed to an up-regulation of xenobiotic metabolism and a down regulation of pro-inflammatory pathways, mediated by PXR activation of RXR^33^. The farnesoid X receptor, together with RXR, plays a crucial role in linking bile acid regulation with lipoprotein, lipid, and glucose metabolism. FXR activation has also been shown to limit the inflammatory response in the gut^34^. Taken together, the activation of these receptors is known to play a significant role in the control of numerous metabolic pathways and anti-inflammatory processes in the gut.

A third relevant theme apparent from the slow response gene expression profile involves a number of pathways that converge in the mitochondria and impact mitochondrial function. This is highly significant since mitochondrial function serves as a key regulator of intestinal epithelial cell homeostasis, and alterations in mitochondrial function are associated with inflammatory bowel disease^35^. The ketogenesis pathway, significantly upregulated in *cluster 2*, describes the synthesis of acetoacetate, D-2-hydroxybutyrate and acetone – the ketone bodies. Ketolysis, also upregulated in *cluster 2*, is the pathway whereby the ketone bodies are converted to acetyl-CoA, which then feeds into the TCA cycle and oxidative phosphorylation for the generation of ATP in the mitochondria. A significant number of transcripts in each of these three pathways are upregulated in the slow responder group. The canonical pathway *mitochondrial dysfunction*, into which each of the other three pathways feed, includes 16 DETS from *cluster 2*, of which 14 are upregulated in slow responders. The upregulated genes are active in three of the four mitochondrial complexes as follows: (a) **complex I**: NADH dehydrogenase, subunit 4L (ND4L), (b) **complex III**: cytochrome b (CYB) and ubiquitol-cytchrome c reductase core protein 1 (UQCRC1) and (c) **complex IV**: cytochrome c oxidase subunit I (COX1), subunit II (COX2), subunit 5B (COX5B), subunit 6A1 (COX6A1) and subunit 6B2 (COX6B2).

Mitochondrial dysfunction is known to be associated with inflammatory and neoplastic pathologies in the gastrointestinal tract^35^. Recently, mitochondrial dysfunction has also been reported to occur at a significant rate in children with ASD. Although the exact etiology is unclear, the clinical aspects of mitochondrial dysfunction in children with ASD include neurodevelopmental regression, gastrointestinal symptoms, seizures, motor delays, fatigue, and lethargy^36^.

Our data are consistent with a recent report of cecal, but not rectal, mitochondrial dysfunction in GI-symptomatic children with ASD^28^ that is not present in inflamed intestinal mucosa of TD children with Crohn’s disease. Interestingly, this same study reported dysbiosis in the right colon, but nowhere else, in the GI-symptomatic children with ASD. Another finding relevant to the current study was mitochondrial over-activity of complex I, III, IV in the cecum of children with ASD, consistent with our data reported here, that found upregulated expression of genes in each of these three mitochondrial complexes in the right colon of the slow responder group. The GI mucosal markers of mitochondrial dysfunction present in children with ASD and intestinal inflammation, and the absence of mitochondrial dysfunction markers in the GI mucosa of TD children with Crohn’s disease, is consistent with differences in the cellular and molecular characteristics of the intestinal inflammation of these two disorders and may explain the persistence of regional right sided fecal loading in patients with ASD.

From a clinical and mechanistic standpoint, our data suggest that in the slow responder group, initial anti-inflammatory therapy achieved resolution of all presenting GI symptoms with the exception of right sided fecal loading. The mechanism may be the result of an intrinsic, perhaps genetic, susceptibility of metabolic/mitochondrial integrity of the colon in the face of a pro-inflammatory cytokine storm. This could render the mitochondria dysfunctional, and thus impairing the function of the involved region of the bowel. By necessity, such a proposition would require either a greater or more sustained pro-inflammatory milieu in the region of the cecum/ascending colon, or conversely, a more highly susceptible mitochondrial population to the effects of this localized pro-inflammatory environment. The fact that the children in the slow responder group in this study were significantly older than the children in the fast responder group when they were being treated for their constipation, and may have harbored symptoms for a longer time prior to diagnosis and treatment, raises the possibility that a longer period of chronic inflammation may influence the response to therapy timeline. Our data also cannot rule out the possibility of a group of children whose mitochondria are more susceptible and/or slower to recover from inflammatory insult.

In further support of these proposed mechanisms, the transcript coding for tumor necrosis factor alpha (TNF-α; a pro-inflammatory cytokine of pathogenic importance in Crohn’s disease), as well as a number of additional tumor necrosis factor-related transcripts, has been shown to be present in excess in the inflamed intestinal mucosa as well as in their peripheral lymphocytes^26,27^ of GI symptomatic children with ASD. In the present study, a large number of transcripts coding for tumor necrosis factor ligands (TNFSF8, TNFSF11, TNFSF13B, TNFSF14), receptors (TNFRSF1B, TNFRSF4, TNFRSF6B, TNFRSF8, TNFRSF9, TNFRSF11B, TNFRSF13B, TNFRSF13C), and induced proteins (TNFAIP1 and TNFAIP8) are all expressed at a significantly higher level in the fast responding group compared to the slow responders. TNF-α is also known to be a particularly toxic agent to functional mitochondria, rendering them dysfunctional in the setting of the “cytokine storm” of inflammatory activity. Thus, it is entirely plausible that TNF-α is functioning as a mitochondrial toxin in the setting of the unique inflammatory characteristics of ASD intestinal inflammation, resulting in decreased energy production and activity of the affected regional tissue. The fact that the right-sided fecal loading in the slow responding patients will eventually resolve over time with sustained anti-inflammatory therapy suggests that mucosal recovery in these patients takes significantly longer than in fast responders.

Taken together, the gene expression data demonstrate that the most prominent molecular processes of the fast responder cluster are those of activation of pro-inflammatory pathways and T cell activity, whereas, the most prominent molecular processes of patients in the slow responder cluster are those that converge to significantly reduce colonic motility. From the standpoint of translational medicine and treatment, this could help to explain why patients in *cluster one* showed resolution of hypomotility along with resolution of their other presenting GI symptoms with administration of anti-inflammatory medication whereas patients in *cluster two* did not experience a resolution of right sided fecal loading in tandem with their other presenting GI symptoms.

The findings outlined in this patient population with ASD may provide direction when considering the larger and more common finding of chronic constipation in infants and children. Many of these children are diagnosed with “idiopathic constipation” because they lack demonstrable pathology and are therefore typically treated symptomatically. Most will improve over months to years. However, it is probably not reasonable to assume a “functional” etiology in all, or one that is somehow psychologic in origin^37^. Rather, consideration should be given to the possibility of a defined and perhaps treatable etiology where the mechanism has not yet been fully investigated.

Chronic constipation has been shown to result from chronic inflammation such as in Chagas disease and has also been shown to be associated with certain peptide deficiencies such as motilin and substance P^37^. It has also been observed that children, following corrective surgery for Hirschprung’s disease, can present with fecal retention more focused in the right colon in a similar pattern to that seen in many of these patients with autism^38^. Additional studies that would be helpful in further elucidating the nature of chronic constipation in ASD would involve analysis of the vagally-mediated enteric nervous system, the innate enteric nervous system, the neuromuscular synaptic transmission, and the integrity of the intestinal smooth muscle. Any or all of these mechanisms may play a role in constipation in children with ASD, and likely contain pathology that complements that which we have described here. In addition, the complete regional mapping of the ASD colonic mucosa in terms of its cellular, immunohistochemical, molecular, and genomic makeup will likely lead to further translational understanding and therapeutic interventions. This approach is supported by the observation that regional involvement of the colon is present in many diseases such as ulcerative colitis, Crohn’s disease, and Hirschsprung’s disease, and their regional patterns of distribution have offered clues in the understanding of their pathophysiology.

Perhaps one of the most significant findings to come out of this pilot study is a putative biomarker that may be able to predict, prior to treatment, which patients with chronic constipation will respond and which patients will be refractory to anti-inflammatory therapy. Within the large number of differentially expressed genes found in the comparison between samples in *cluster 1* and *cluster 2*, there is a single long-noncoding RNA (lncRNA), Xxbac-B476C20.9, whose gene expression profile was able to predict sample assignment to *clusters 1 and 2* with 89% accuracy (88% sensitivity and 89% specificity). This same lncRNA transcript was recently reported to be one of six lncRNAs comprising a biomarker signature with promising potential for predicting survival in colorectal cancer patients^39^.

Long non-coding RNAs are transcripts, longer than 200 nucleotides, which are not translated into protein. Current estimates suggest that there are tens of thousands of these lncRNAs in the human genome. These lncRNAs play an important role in a diverse range of biological phenomena^40^, such as imprinting and chromosomal conformation^41^, coordination of cell state and differentiation^42,43^, enzymatic regulation^44^, and disease^45^. Thus, it is not unreasonable to believe that lncRNA could play a role in ASD – including both having a functional role and being predictive. Both the overexpression and the deficiency of lncRNAs have been implicated in a number of human diseases^40^. In general, transcriptome-wide studies have shown that lncRNAs exhibit more specific expression profiles than mRNAs, suggesting that lncRNA expression is even more tightly regulated than that of protein-coding genes^40^.

While the lncRNA suggested here may be co-expressed with a number of protein-coding genes, the LASSO regression is identifying it as the single most important predictor of response for a reason. This may be a result of tighter regulation of this lncRNA which has minimized the variance of expression of this lncRNA, thereby making it a better predictor by increasing the power to detect differences in expression of the lncRNA over other transcripts. These results are both important in and of themselves, but the lncRNA form an intriguing hypothesis that needs to be tested in larger cohorts. One practical implication of these data could be advocating a “top down” treatment approach in patients identified as slow responders and a “bottom up” approach to those identified as fast responders, but it will first be necessary to perform additional, well powered studies to test the predictive ability of Xxbac-B476C20.9 and/or any additional putative biomarkers.

This study represents the first attempt to explore the molecular basis of right-sided colonic dysmotility in GI-symptomatic children with ASD. In addition to the obviously modest number of samples in each of the two clinical groups, another limitation of this study is the disparity in the average age between the fast (younger) and slow (older) responders. The original analysis was performed without correcting for age and then the final analysis was performed to account for this statistically significant age difference and although the number and composition of the differentially expressed genes changed somewhat, the overall biological themes did not. We suspect that the difference in age, rather than being a chance occurrence, may actually reflect a biologically relevant phenomenon; i.e. it is possible that children who have lived with an undiagnosed chronic gastrointestinal inflammation for a longer period of time before being seen by the gastroenterologist will perhaps be slower to respond to therapy.

## Conclusions

In this study, we present gene expression data that distinguishes ASD fast response constipation from ASD persistent right-sided constipation. Of greater value, the molecular pathways reflected in these gene expression profiles provide insight into differing underlying pathologic mechanisms of these two groups that impact the response to therapy. Moreover, we have identified a sensitive and specific biomarker that can reliably predict the clinical course of a given patient with ASD, GI symptoms, and constipation. This finding may have significant implications for the management of patients with ASD seen in the pediatric gastroenterology clinic. Work to validate these findings in a second cohort is currently underway.

## Methods

### Patient selection and enrollment

Participants for this study were chosen based on a retrospective chart review from a single private-practice pediatric gastroenterology clinic. Patients referred for pediatric gastroenterologic evaluation had chronic gastrointestinal symptoms and a previously confirmed diagnosis of ASD based upon DSM-IV criteria and/or ASD-specific quantitative metrics. Inclusion criteria for this study were: (a) confirmed diagnosis of ASD at the time of gastrointestinal evaluation, (b) chronic constipation, either isolated or in association with other chronic gastrointestinal symptoms, (c) a diagnosis of enteritis, colitis, or enterocolitis based on histopathology and/or capsule endoscopy, (d) pediatric age (between 1–21 years old), and (e) either a *fast* (within 4–8 weeks) and sustained resolution of signs and symptoms of constipation with maintenance anti-inflammatory medication and infrequent need for laxatives, or *slow* (requiring regular bowel cleanouts for up to 3 years) response to anti-inflammatory therapy and a regular need for laxatives. In addition to chronic constipation, commonly associated symptoms included abdominal pain, diarrhea, fecal incontinence, abdominal distention, rumination, and failure to thrive, in addition to abnormal stool descriptors such as light colored and malodorous stools. In all cases, non-invasive evaluation of blood, stool, and urine, in addition to abdominal radiography, failed to provide a diagnosis.

Chronicity of the constipation at presentation, as assessed by the treating gastroenterologist (AK), was similar amongst all patients, including the relative prevalence of constipation-only and alternating constipation/diarrhea. Three slow responder patients and one fast responder patient were taking either psychotropic medication and/or anti-convulsants, (e.g. memantine, clobazam, zonisamide, guanfacine) that are associated infrequently with constipation. In all but one patient (a slow responder) the medications were discontinued during treatment for enterocolitis and constipation without an observed effect on the need for chronic laxatives.

Following standard pre-procedure bowel prep, each patient underwent clinically indicated diagnostic ileocolonoscopy, esophagogastroduodenoscopy (EGD) and biopsy, and capsule endoscopy. All study participants were found to have non-specific mucosal inflammation of the small or large intestine (detected on routine hematoxylin and eosin (H&E) staining, capsule endoscopy, or both), in the absence of any other identifiable pathology, and were treated with an initial course of oral corticosteroids. At this point the clinical course diverged and revealed two distinct patient populations. One group (slow responding chronic constipation) demonstrated clinical relief from associated symptoms such as diarrhea and abdominal pain but continued to experience recurrent episodes of symptomatic constipation and right sided fecal loading, not attributable to voluntary withholding behavior, which required regularly scheduled bowel cleanouts in addition to oral non-steroidal anti-inflammatory therapy. The duration of the need for regularly scheduled cleanouts varied from 1 to 3 years. The other group (fast responding chronic constipation) demonstrated not only clinical relief of associated symptoms but also resolution of their chronic constipation while on maintenance non-steroidal anti-inflammatory therapy.

### Sample preparation, RNA isolation, RNA-sequencing

Right colonic mucosal biopsy tissue was collected during ileocolonoscopy and placed immediately in RNAlater upon procurement. Total ribonucleic acid (RNA) was isolated from the biopsy tissue using a QIAcube robotic workstation with reagents and protocols following manufacturer recommendations (Qiagen, Valencia, CA). Total RNA was analyzed for quantity (Nanodrop spectrophotometer) and quality (Agilent Bioanalyzer). A single bowel biopsy specimen, typically 3–5 mg of tissue, routinely yielded 3–5 µg high quality (RIN ≥ 7) total RNA. RNA sequencing (RNAseq) was performed on an Illumina NextSeq500 NGS at the Cancer Genomics Shared Resource Facility, Wake Forest Health Sciences.

### Estimating gene expression and differential gene expression analysis

Raw sequences were examined for quality using FASTQC, and the alignment of reads was performed using STAR two pass alignment^46^. Gene counts were determined using the program featureCounts^47^ and DESeq2^48^ was used to normalize gene counts and find differentially expressed genes while adjusting for age and sex. Differentially expressed transcripts (DETs) that met an average read count threshold >5, had a false discovery rate (FDR) adjusted p-value < 0.05, and a fold change >1.3 or <0.77 (i.e., 1/1.3) were retained for further evaluation. To generate biologic context and hypotheses for the DET (P < 0.001), pathway analysis and identification of upstream regulators of the associated genes were completed using Ingenuity Pathway Analysis (IPA).

### Hierarchical clustering analysis

Unsupervised hierarchical cluster analysis on the DETs that met the P < 0.001 threshold was computed using Euclidean distances between the normalized expression values for each of the samples. In hierarchical clustering, each sample is assigned to its own cluster and the algorithm then iteratively joins the two closest clusters at each stage until all samples are joined to a single cluster. The number of clusters was determined by the Bayesian information criterion (BIC) or Schwarz criterion. A dendrogram and DET heatmap were generated to display the resulting cluster patterns.

### Predicting cluster assignment

As was discussed in the Results section, two clusters were empirically identified by the BIC. A penalized regression (LASSO) under a logistic model was used to identify the genes whose transcript counts (expression levels) were most predictive of membership within one of the two cluster assignments. Penalized regression analysis is a statistical machine-learning tool specifically designed to identify the key predictors from a set of predictors (e.g., gene expression values) that greatly outnumber the number of subjects by shrinking the regression coefficients of non-important predictors to zero. The value of the LASSO penalty parameter (λ), which controls the regression parameters degree of shrinkage, was estimated as the value of λ that minimized the misclassification error rate using ten-fold cross validation. Next, the set of genes with non-zero parameter estimates was used for predicting cluster assignment and the logistic model for clusters 1 and 2 was recomputed to obtain predicted values for each individual using a leave-one-out strategy. Specifically, for the i^th^ individual from the combined sample of clusters 1 and 2, a logistic model was computed for cluster assignment leaving out the ith individual. From this model, the regression coefficients were then applied to the i^th^ individual to obtain an independent prediction of its classification. The process was repeated 35 times, one time for each individual, and the resulting confusion matrix (2 × 2 table of predicted versus observed values) was computed. To summarize the predictive ability of these models, the sensitivity (probability that an individual will have a predicted classification of fast responder when the actual classification is fast responder), specificity (probability that an individual will have a predicted classification of slow responder if the actual classification is slow responder) and Cohen’s kappa statistics were computed. The kappa statistic, ranging from −1 (complete disagreement) to 1 (complete agreement), measures the agreement between the predicted and actual classifications, accounting for the amount of agreement that should occur by change alone.

### Ethics approval and consent to participate

All research was performed in accordance with relevant guidelines/regulations and informed consent was obtained from the parent or caregiver for all individual participants included in this study under a research protocol approved by the Copernicus Group Institutional Review Board (IRB# WF-11-081).

## Supplementary information

Supplementary Information

## Data Availability

The datasets generated and/or analyzed during the current study are not publicly available at this time but will be deposited into GEO (https://www.ncbi.nlm.nih.gov/geo/*)* upon acceptance of the manuscript for publication. The data are available at any time from the corresponding author on reasonable request.
