# Supplementary material for: A molecular biomarker for prediction of clinical outcome in children with ASD, constipation, and intestinal inflammation
Source: Sci Rep. 2019 Apr 12;9:5987. doi: 10.1038/s41598-019-42568-1 (PMC6461625; doi:10.1038/s41598-019-42568-1)
Supplement: Supplementary file 1 — Supplementary Information [file 41598_2019_42568_MOESM1_ESM.pdf]

A molecular biomarker for prediction of clinical outcome in children with ASD, constipation, and intestinal inflammation

Stephen J Walker, Carl D Langefeld, Kip Zimmerman, Marshall Z Schwartz, Arthur Krigsman

Additional Files

Additional File 1: Differentially-expressed transcripts in colonic mucosa between slow responders and fast responders.

Additional File 2: Xxbac-B476C20.9 expression in colon and ileum.

Additional File 1 - Differentially expressed transcripts in colonic mucosa between slow responders and fast responders

EnsembleID	GeneSymbol	log2FoldChange	FoldChange	P-Value	P-Adj	log(P)
ENSG00000267369	RP11-1094M14.8	-0.702104851	0.614674759	3.12E-06	0.025453221	5.505845406
ENSG00000232810	TNF	-0.647294951	0.638476333	9.28E-05	NA	4.032452024
ENSG00000231102	RP11-298J23.5	-0.62513039	0.648361176	2.41E-05	0.050330304	4.617982957
ENSG00000235117	RP11-229P13.20	-0.612703438	0.653970091	0.000125974	NA	3.89971908
ENSG00000213434	VTI1BP2	-0.598490215	0.660444751	0.000330072	NA	3.481391315
ENSG00000223345	HIST2H2BA	-0.591060378	0.663854796	0.000402569	NA	3.395159671
ENSG00000201794	RN7SKP130	-0.590981588	0.663891052	0.00026536	NA	3.576164541
ENSG00000231466	CTA-246H3.11	-0.588820063	0.664886475	9.74E-06	0.025453221	5.011441043
ENSG00000142233	NTN5	-0.586373141	0.666015131	0.000422829	NA	3.373835234
ENSG00000140285	FGF7	-0.578158876	0.669818034	0.000160506	0.095899974	3.794508728
ENSG00000254656	RTL1	-0.574952069	0.671308554	0.00039568	NA	3.402655901
ENSG00000225146	AC073957.15	-0.568068712	0.674519141	0.00057422	NA	3.240921685
ENSG00000214975	PPIAP29	-0.560014856	0.678295179	0.00075151	NA	3.124065236
ENSG00000226380	MIR29B1	-0.556495598	0.679951807	6.47E-06	0.025453221	5.189095719
ENSG00000258581	RP11-638I2.10	-0.545510569	0.685148886	0.000874406	NA	3.058286871
ENSG00000143185	XCL2	-0.540104603	0.687721044	0.000736287	NA	3.132952867
ENSG00000227083	L29074.3	-0.539825487	0.687854109	0.000438526	NA	3.358004652
ENSG00000019991	HGF	-0.539420339	0.688047304	0.000846236	0.126745464	3.072508503
ENSG00000145777	TSLP	-0.535547814	0.689896662	0.000854586	0.126745464	3.068244226
ENSG00000240007	RP6-206I17.4	-0.535424327	0.689955716	0.000912814	NA	3.039617708
ENSG00000267671	RP11-115K3.2	-0.535326762	0.690002377	0.000570659	NA	3.243623329
ENSG00000157168	NRG1	-0.529736975	0.692681009	0.00033224	0.104657875	3.478548082
ENSG00000255987	RP11-1094M14.4	-0.529004424	0.693032818	0.00096555	0.133558216	3.015225232
ENSG00000198155	ZNF876P	-0.522829264	0.69600556	0.000358124	0.106870746	3.445966573
ENSG00000226440	RP11-506B6.6	-0.522164625	0.696326278	0.000570276	NA	3.243914905
ENSG00000155918	RAET1L	-0.508310052	0.703045491	0.000237783	NA	3.623819198
ENSG00000146592	CREB5	-0.503288058	0.705497043	0.000624865	0.118792513	3.2042138
ENSG00000171094	ALK	-0.502856371	0.705708176	0.000891911	0.13043101	3.04967848

ENSG00000232677	LINC00665	-0.497910861	0.708131471	0.000563055	0.114740192	3.249449181
ENSG00000183386	FHL3	-0.492712051	0.710687854	0.000121161	0.087369445	3.916637151
ENSG00000232110	RP11-149I23.3	-0.485062417	0.714466158	0.000479722	NA	3.319010364
ENSG00000160716	CHRNB2	-0.477128587	0.718406054	6.37E-06	0.025453221	5.195860568
ENSG00000227413	RP5-1042K10.12	-0.471103234	0.72141272	0.000826472	0.126154599	3.082771855
ENSG00000253919	PRKRIRP7	-0.467064509	0.723435094	2.80E-05	0.051009747	4.552841969
ENSG00000227006	RP5-956O18.2	-0.459486617	0.727245003	0.000580686	0.114921083	3.236058644
ENSG00000213197	AC012066.1	-0.449869244	0.732109198	0.00014719	0.090829249	3.832121695
ENSG00000104728	ARHGEF10	-0.448555441	0.732776204	3.13E-05	0.051009747	4.504455662
ENSG00000261528	AC002400.1	-0.446082691	0.734033244	0.000866134	0.127553495	3.062414913
ENSG00000228196	PTPN2P1	-0.444452097	0.734863348	0.000135875	0.090829249	3.866860443
ENSG00000171790	SLFNL1	-0.433311381	0.740560045	0.000245432	0.10305307	3.610068814
ENSG00000254317	RP11-473O4.5	-0.431675896	0.741400043	0.000403583	0.111048967	3.394067136
ENSG00000248476	BACH1-IT1	-0.41995986	0.74744542	0.000310623	0.104657875	3.50776639
ENSG00000120694	HSPH1	-0.416770232	0.749099762	0.000925744	0.132596927	3.033509094
ENSG00000240731	RP5-890O3.9	-0.405098251	0.755184861	0.000388036	0.109656921	3.411127981
ENSG00000236194	AC003104.1	-0.391111739	0.762541764	0.000401415	0.111048967	3.396406403
ENSG00000224997	AL049840.1	-0.38264089	0.767032233	0.000528364	0.114740192	3.277066781
ENSG00000175564	UCP3	-0.364603376	0.77668237	0.000914847	0.131939809	3.038651532
ENSG00000255220	DDX18P5	-0.360082448	0.779120053	0.000791325	0.125163736	3.101645114
ENSG00000134470	IL15RA	-0.350951327	0.784066906	0.000434815	0.11108458	3.361695482
ENSG00000219665	CTD-2006C1.2	-0.346777234	0.786338701	0.000286958	0.104657875	3.542181663
ENSG00000246422	CTD-2024I7.13	-0.345226998	0.787184109	0.000340318	0.104657875	3.46811508
ENSG00000180089	TMEM86B	-0.340670202	0.789674384	0.000338316	0.104657875	3.470677463
ENSG00000261355	RP11-698N11.4	-0.337641149	0.79133411	0.000167277	0.097169092	3.776563769
ENSG00000166246	C16orf71	-0.337488024	0.791418106	0.000372967	0.108326299	3.428329593
ENSG00000256690	RP11-727F15.9	-0.334391175	0.793118766	4.62E-05	0.060372026	4.335358024
ENSG00000134013	LOXL2	-0.33145016	0.794737233	0.00069806	0.122803898	3.156107247
ENSG00000269808		-0.328740068	0.796231544	0.000595078	0.115224761	3.225426105
ENSG00000260772	RP11-311C24.1	-0.275620227	0.826095096	5.35E-05	0.065819972	4.271646218
ENSG00000165632	TAF3	-0.272136548	0.82809228	7.27E-05	0.071239686	4.138465589

ENSG00000171574	ZNF584	-0.271932742	0.828209271	0.000759806	0.125163736	3.119297281
ENSG00000065526	SPEN	-0.271364056	0.828535802	0.000241328	0.10305307	3.617392286
ENSG00000175471	MCTP1	-0.256123086	0.837335046	0.000424634	0.11108458	3.371985235
ENSG00000184675	AMER1	-0.24676565	0.842783719	0.000309307	0.104657875	3.509610251
ENSG00000142207	URB1	-0.243626232	0.84461968	0.000762578	0.125163736	3.117715728
ENSG00000127947	PTPN12	-0.241143288	0.846074561	0.000319279	0.104657875	3.495829645
ENSG00000102908	NFAT5	-0.23014906	0.852546802	1.10E-05	0.025488908	4.958607315
ENSG00000126215	XRCC3	-0.222979967	0.856793852	0.00024473	0.10305307	3.61131279
ENSG00000157827	FMNL2	-0.211829361	0.863441679	7.49E-05	0.071239686	4.125518182
ENSG00000197024	ZNF398	-0.209077998	0.865089919	6.36E-05	0.070047491	4.196542884
ENSG00000269958	RP11-73M18.8	-0.200309201	0.870364005	0.000483323	0.114740192	3.315762538
ENSG00000138668	HNRNPD	-0.198752991	0.871303358	6.79E-05	0.071005586	4.168130226
ENSG00000075407	ZNF37A	-0.175520223	0.885448177	0.000205515	0.099731231	3.687156475
ENSG00000170144	HNRNPA3	-0.162801867	0.893288524	0.0009867	0.133558216	3.005814872
ENSG00000011523	CEP68	-0.147845732	0.902597237	0.000450731	0.112210653	3.346082571
ENSG00000177646	ACAD9	-0.147807471	0.902621175	0.000494727	0.114740192	3.305634387
ENSG00000115216	NRBP1	0.176045605	1.129782928	0.000683668	0.122803898	3.165154747
ENSG00000177156	TALDO1	0.188201087	1.139342171	0.000980424	0.133558216	3.008586066
ENSG00000100938	GMPR2	0.19207172	1.142403037	0.000582519	0.114921083	3.234689905
ENSG00000158604	TMED4	0.193600843	1.14361452	0.000321779	0.104657875	3.492442302
ENSG00000162384	C1orf123	0.201264599	1.149705692	0.000362845	0.106870746	3.440278857
ENSG00000112167	SAYSD1	0.203930632	1.151832258	0.000671712	0.122803898	3.172816893
ENSG00000181192	DHTKD1	0.218294696	1.163357654	8.81E-05	0.073187151	4.055024092
ENSG00000205744	DENND1C	0.219155743	1.16405219	0.000147676	0.090829249	3.83069008
ENSG00000229358	DPY19L1P1	0.221529734	1.165969244	0.000975688	0.133558216	3.010689036
ENSG00000117448	AKR1A1	0.224032284	1.167993531	3.41E-05	0.051009747	4.467245621
ENSG00000085760	MTIF2	0.233329348	1.175544659	0.000796664	0.125163736	3.098724807
ENSG00000117242	PINK1-AS	0.234356925	1.176382253	0.0005129	0.114740192	3.289967301
ENSG00000249884	RNF103-CHMP3	0.235255777	1.177115411	0.000813995	0.125163736	3.089378263
ENSG00000166946	CCNDBP1	0.237007523	1.178545553	0.000668899	0.122803898	3.174639453
ENSG00000185825	BCAP31	0.241750098	1.182426164	0.000208761	0.099731231	3.680350631

ENSG00000234608	MAPKAPK5-AS1	0.243709888	1.18403349	0.000135594	0.090829249	3.867759527
ENSG00000117614	SYF2	0.247331988	1.187009917	0.000339288	0.104657875	3.4694315
ENSG00000117450	PRDX1	0.252296713	1.191101793	0.00081146	0.125163736	3.090732883
ENSG00000162441	LZIC	0.252533714	1.191297479	0.000356449	0.106870746	3.448002599
ENSG00000205808	PPAPDC2	0.270716987	1.206407236	0.000996322	0.133558216	3.00160028
ENSG00000248594	AC003029.1	0.276199655	1.211000663	0.00093496	0.133005971	3.029206969
ENSG00000140374	ETFA	0.278562293	1.21298549	0.000547841	0.114740192	3.261345469
ENSG00000100577	GSTZ1	0.282144004	1.216000653	0.000527689	0.114740192	3.277621959
ENSG00000145545	SRD5A1	0.286755102	1.219893415	0.000508495	0.114740192	3.293713313
ENSG00000132313	MRPL35	0.2905787	1.223130807	0.000142351	0.090829249	3.846639478
ENSG00000153774	CFDP1	0.294487465	1.226449189	0.000751622	0.125163736	3.124000517
ENSG00000181826	RELL1	0.295166491	1.227026571	0.000746256	0.125163736	3.127112164
ENSG00000069849	ATP1B3	0.296183508	1.227891859	0.000337707	0.104657875	3.471459937
ENSG00000167130	DOLPP1	0.299762729	1.230941951	0.000553346	0.114740192	3.257003225
ENSG00000131165	CHMP1A	0.300140594	1.231264397	0.000107286	0.080127668	3.969456946
ENSG00000101417	PXMP4	0.300301994	1.231402151	0.000663589	0.122803898	3.178100822
ENSG00000178537	SLC25A20	0.300843876	1.231864758	0.000622028	0.118792513	3.206190066
ENSG00000060642	PIGV	0.303558174	1.234184579	0.000208271	0.099731231	3.681371198
ENSG00000138794	CASP6	0.306237122	1.236478472	0.000992896	0.133558216	3.003096239
ENSG00000271092	RP11-57H12.6	0.309786022	1.239523842	0.000380747	0.109070986	3.41936351
ENSG00000177042	TMEM80	0.312584112	1.241930217	0.000285281	0.104657875	3.544727152
ENSG00000124523	SIRT5	0.314490761	1.243572623	0.00020984	0.099731231	3.678111722
ENSG00000164010	ERMAP	0.314741152	1.243788473	0.000251325	0.10305307	3.599764309
ENSG00000102078	SLC25A14	0.31930346	1.247727994	9.10E-05	0.073187151	4.040958608
ENSG00000123159	GIPC1	0.319451561	1.247856088	0.00043489	0.11108458	3.361620579
ENSG00000104886	PLEKHJ1	0.320185972	1.248491477	0.000710421	0.122803898	3.148484209
ENSG00000228192	RP11-342M1.3	0.328947285	1.256096483	0.000754231	0.125163736	3.122495621
ENSG00000213977	TAX1BP3	0.333803391	1.260331623	0.000703588	0.122803898	3.152681576
ENSG00000242797	GLYCTK-AS1	0.336926556	1.263062961	0.00078633	0.125163736	3.104395155
ENSG00000130517	PGPEP1	0.337838549	1.263861653	8.34E-05	0.073187151	4.078833949
ENSG00000160325	CACFD1	0.347761345	1.272584403	0.000179994	0.099053811	3.744741972

ENSG00000128311	TST	0.351870057	1.276213816	0.000498475	0.114740192	3.302356618
ENSG00000187210	GCNT1	0.352321714	1.276613416	0.000977475	0.133558216	3.009894341
ENSG00000232935	RP11-223P11.2	0.354631416	1.278658864	0.000209462	0.099731231	3.678894754
ENSG00000168237	GLYCTK	0.356959967	1.280724322	0.000556431	0.114740192	3.254588683
ENSG00000161267	BDH1	0.357883709	1.281544619	0.000654331	0.122803898	3.184202504
ENSG00000225335	XXbac-B476C20.9	0.363957178	1.286951048	0.000230174	0.10305307	3.637943735
ENSG00000117472	TSPAN1	0.365838588	1.288630448	0.000679921	0.122803898	3.167541545
ENSG00000169814	BTD	0.366200834	1.28895405	4.07E-05	0.056808933	4.390405591
ENSG00000243989	ACY1	0.376982466	1.29862282	0.000267577	0.104657875	3.57255122
ENSG00000114786	ABHD14A-ACY1	0.390933579	1.311241644	0.00022629	0.10305307	3.645334638
ENSG00000009765	IYD	0.392616426	1.312772048	0.000813387	0.125163736	3.089702773
ENSG00000166391	MOGAT2	0.403390137	1.322612221	0.000977525	0.133558216	3.009872127
ENSG00000254837	AP001372.2	0.406018819	1.325024301	0.000318049	0.104657875	3.497505966
ENSG00000162433	AK4	0.407021408	1.325945436	0.00074978	0.125163736	3.125066148
ENSG00000167117	LINC00483	0.417153643	1.3352905	0.000710562	0.122803898	3.148398022
ENSG00000178226	PRSS36	0.420393436	1.338292469	0.000473031	0.114740192	3.325110397
ENSG00000132359	RAP1GAP2	0.420984033	1.338840439	0.000952136	0.133558216	3.021301014
ENSG00000187699	C2orf88	0.423616887	1.341285994	0.000271225	0.104657875	3.566670282
ENSG00000229155	RP11-528A4.2	0.427491579	1.34489317	0.000687939	0.122803898	3.162450069
ENSG00000135324	MRAP2	0.428763933	1.346079793	0.000248723	0.10305307	3.604284053
ENSG00000272086	CTD-2186M15.3	0.431417067	1.348557528	0.00053708	0.114740192	3.26996102
ENSG00000224259	RP11-48O20.4	0.437106555	1.353886271	0.000311243	0.104657875	3.506900407
ENSG00000156006	NAT2	0.440147082	1.35674264	9.63E-05	0.07454916	4.016373713
ENSG00000187824	TMEM220	0.4568907	1.372580439	0.000850189	0.126745464	3.070484518
ENSG00000214796	RP11-480I12.5	0.457852902	1.373496184	0.000569702	0.114740192	3.244352256
ENSG00000249267	LINC00939	0.459994204	1.375536292	0.000476361	NA	3.322063802
ENSG00000213302	RP11-560I19.2	0.469689662	1.384811549	0.000907017	NA	3.042384573
ENSG00000103260	METRN	0.47465095	1.38958198	0.000294093	0.104657875	3.531515312
ENSG00000241224	RP11-59E19.1	0.492583386	1.406962021	0.000440896	0.11108458	3.355663841
ENSG00000172159	FRMD3	0.496261368	1.410553482	8.68E-05	0.073187151	4.061480275
ENSG00000228630	HOTAIR	0.500571724	1.414774111	0.000339869	NA	3.468688446

ENSG00000250567	CTD-2154H6.1	0.5083485	1.422420972	0.000836297	NA	3.077639461
ENSG00000231887	PRH1	0.509096217	1.423158372	0.000854294	0.126745464	3.068392644
ENSG00000158486	DNAH3	0.510564091	1.424607105	0.000772795	0.125163736	3.111935696
ENSG00000265933	LINC00668	0.516902849	1.430880161	0.000913764	0.131939809	3.039165956
ENSG00000264127	SCML2P1	0.523354997	1.437293801	0.000786943	0.125163736	3.104056723
ENSG00000181019	NQO1	0.526844015	1.440773963	0.0004993	0.114740192	3.301638434
ENSG00000214264	RP11-356J5.5	0.530617199	1.444547055	0.000225276	NA	3.647285074
ENSG00000203306	AP001007.1	0.532826122	1.446760507	0.000766955	0.125163736	3.115230117
ENSG00000266949	LYPD8	0.53892195	1.452886446	0.000329401	0.104657875	3.482275087
ENSG00000267385	CTB-50L17.14	0.539760492	1.453731157	0.000559696	NA	3.252047797
ENSG00000233215	AP000472.2	0.54247436	1.456468359	0.000591305	0.115224761	3.228188449
ENSG00000261602	CTD-2033A16.1	0.54325599	1.457257665	0.0005573	0.114740192	3.253910957
ENSG00000134955	SLC37A2	0.552340256	1.466462578	0.000570628	0.114740192	3.243646922
ENSG00000170298	LGALS9B	0.554786382	1.468951111	0.000431295	0.11108458	3.365225577
ENSG00000213996	TM6SF2	0.560580758	1.474862805	0.000514261	0.114740192	3.28881641
ENSG00000169347	GP2	0.562941241	1.477277895	0.00053171	0.114740192	3.274325172
ENSG00000267629	AC138430.4	0.568933823	1.483426887	0.00043966	0.11108458	3.356883044
ENSG00000210176	MT-TH	0.572029932	1.486613827	5.99E-05	0.06962657	4.222573178
ENSG00000147257	GPC3	0.578952421	1.493764193	0.00046331	0.113985219	3.334128326
ENSG00000187134	AKR1C1	0.579120949	1.493938697	0.000418832	0.11108458	3.377960144
ENSG00000268424	AC008948.1	0.582616847	1.497563157	0.000186422	0.099731231	3.729502837
ENSG00000130600	H19	0.584536015	1.499556639	0.0004168	NA	3.38007229
ENSG00000210184	MT-TS2	0.588499939	1.503682461	9.64E-06	0.025453221	5.015922966
ENSG00000130701	RBBP8NL	0.588680975	1.503871162	3.19E-05	0.051009747	4.496209317
ENSG00000261761	RP11-103J17.2	0.588838121	1.50403498	0.000398362	NA	3.399722096
ENSG00000165192	ASB11	0.589967029	1.505212347	0.000374334	NA	3.426740725
ENSG00000259237	RP11-209E8.1	0.591958998	1.507292071	0.000180822	NA	3.742748732
ENSG00000165188	RNF183	0.60795057	1.524092611	0.000159753	NA	3.796550977
ENSG00000210191	MT-TL2	0.613214527	1.52966372	1.27E-06	0.025453221	5.896196279
ENSG00000247627	MTND4P12	0.625395084	1.54263322	0.000176207	0.099053811	3.753976843
ENSG00000105251	SHD	0.654446756	1.574012236	7.30E-06	0.025453221	5.13667714

ENSG00000184163	FAM132A	0.664945291	1.585508147	8.14E-06	0.025453221	5.089375595
ENSG00000137225	CAPN11	0.667061925	1.587836015	5.97E-05	NA	4.224025669
ENSG00000232175	RP4-659I19.1	0.712128963	1.63821983	2.03E-05	NA	4.692503962

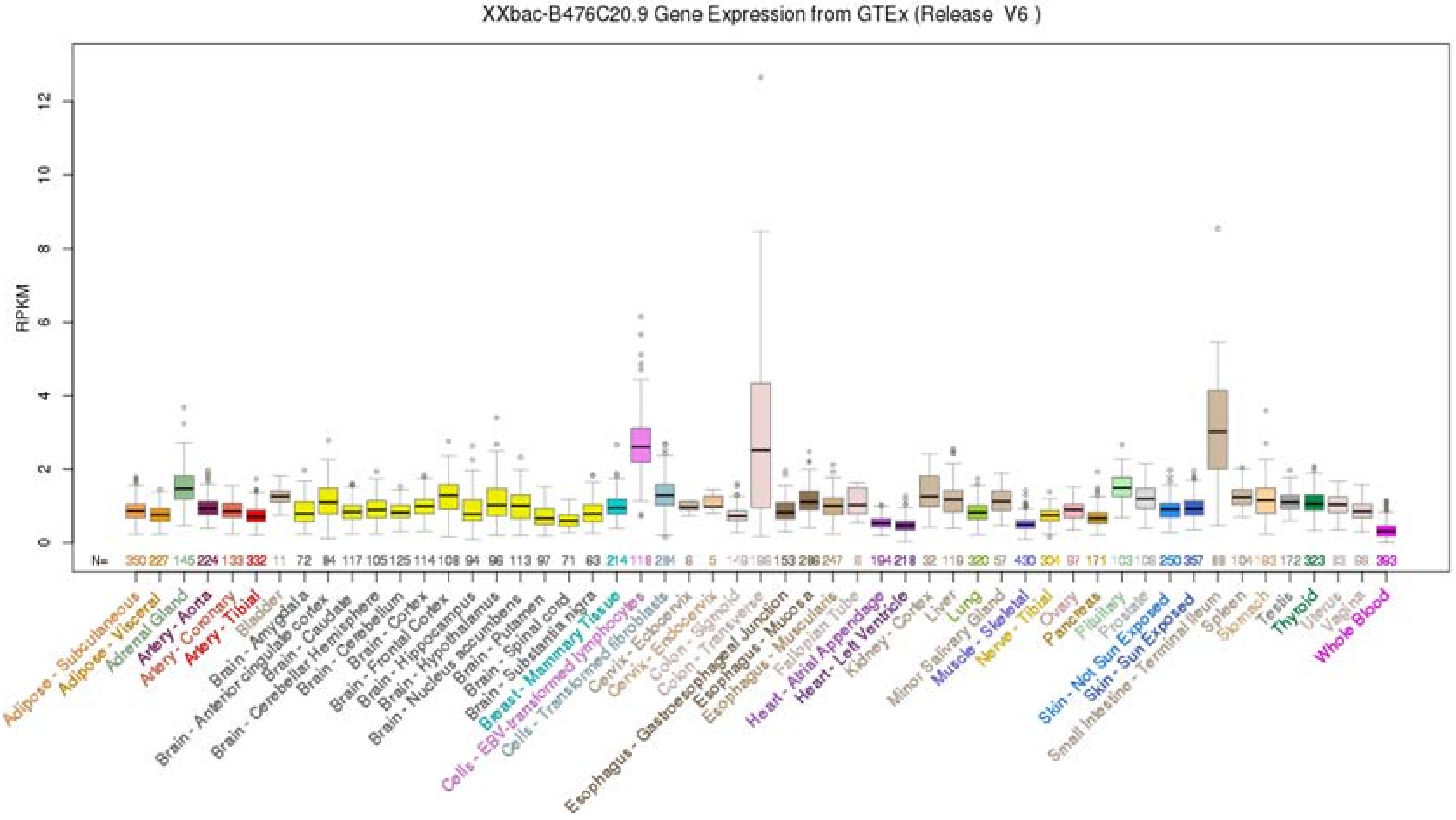

XXbac-B476C20.9 Gene Expression from GTEx (Release V6)
RPKM
0
2
4
6
8
10
12
N=
350 227 145 224 133 332 11 72 84 117 105 125 114 108 94 96 113 97 71 63 214 118 284 6 5 149 196 153 286 247 6 194 218 32 119 320 57 430 304 97 171 103 106 250 357 88 104 163 172 323 83 88 393
Adipose - Subcutaneous
Adipose - Visceral
Adrenal Gland
Artery - Aorta
Artery - Coronary
Artery - Tibial
Bladder
Brain - Amygdala
Brain - Anterior cingulate cortex
Brain - Caudate
Brain - Cerebellar Hemisphere
Brain - Cerebellum
Brain - Cortex
Brain - Frontal Cortex
Brain - Hippocampus
Brain - Hypothalamus
Brain - Nucleus accumbens
Brain - Putamen
Brain - Spinal cord
Brain - Substantia nigra
Breast - Mammary Tissue
Cells - EBV-transformed lymphocytes
Cells - Transformed fibroblasts
Cervix - Ectocervix
Cervix - Endocervix
Colon - Sigmoid
Colon - Transverse
Esophagus - Gastroesophageal Junction
Esophagus - Mucosa
Esophagus - Muscularis
Fallopian Tube
Heart - Atrial Appendage
Heart - Left Ventricle
Kidney - Cortex
Liver
Lung
Minor Salivary Gland
Muscle - Skeletal
Nerve - Tibial
Ovary
Pancreas
Pituitary
Prostate
Skin - Not Sun Exposed
Skin - Sun Exposed
Small Intestine - Terminal Ileum
Spleen
Stomach
Testis
Thyroid
Uterus
Vagina
Whole Blood
